# Non-coding regulatory sRNAs from bacteria of the *Burkholderia cepacia* complex

**DOI:** 10.1007/s00253-024-13121-6

**Published:** 2024-04-02

**Authors:** Gonçalo R. Matos, Joana R. Feliciano, Jorge H. Leitão

**Affiliations:** 1 iBB-Institute for Bioengineering and Biosciences, Instituto Superior Técnico, Universidade de Lisboa, 1049-001 Lisbon, Portugal; 2 Department of Bioengineering, Instituto Superior Técnico, Universidade de Lisboa, 1049-001 Lisbon, Portugal; 3 Associate Laboratory i4HB-Institute for Health and Bioeconomy at Instituto Superior Técnico, Universidade de Lisboa, Av. Rovisco Pais, 1049-001 Lisbon, Portugal

**Keywords:** *Burkholderia cepacia* complex, Small non-coding RNAs, Post-transcriptional regulation

## Abstract

**Abstract:**

Small non-coding RNAs (sRNAs) are key regulators of post-transcriptional gene expression in bacteria. Hundreds of sRNAs have been found using in silico genome analysis and experimentally based approaches in bacteria of the *Burkholderia cepacia* complex (Bcc). However, and despite the hundreds of sRNAs identified so far, the number of functionally characterized sRNAs from these bacteria remains very limited. In this mini-review, we describe the general characteristics of sRNAs and the main mechanisms involved in their action as regulators of post-transcriptional gene expression, as well as the work done so far in the identification and characterization of sRNAs from Bcc. The number of functionally characterized sRNAs from Bcc is expected to increase and to add new knowledge on the biology of these bacteria, leading to novel therapeutic approaches to tackle the infections caused by these opportunistic pathogens, particularly severe among cystic fibrosis patients.

**Key points:**

*•Hundreds of sRNAs have been identified in Burkholderia cepacia complex bacteria (Bcc).*

*•A few sRNAs have been functionally characterized in Bcc.*

*•Functionally characterized Bcc sRNAs play major roles in metabolism, biofilm formation, and virulence.*

## Introduction

Bacterial non-coding RNAs (sRNAs) are now recognized as major post-transcriptional regulators, contributing to a fast adaptation and fine tuning of gene expression to the challenging environmental changes faced by bacteria (Shimoni et al. 2007). This fast adaption and gene expression fine tuning is particularly important in human opportunistic pathogens, as is the case of bacteria of the *Burkholderia cepacia* complex (Bcc). The Bcc comprises at least 26 species, most of them capable of causing severe and often lethal respiratory infections among cystic fibrosis (CF) patients (Martina et al. 2018; Velez et al. 2023). This group of bacteria has received particular attention since the 1980s due to their transmission among CF patients and ability to cause severe and often lethal respiratory infections (Govan et al. 1993). The clinical outcome of Bcc infections is highly variable and strain-dependent, ranging from asymptomatic carriage to the cepacia syndrome, a necrotizing pneumonia often associated with septicemia (Isles et al. 1984; Sousa et al. 2021), with *B. cenocepacia* and *B. multivorans* as the most frequently recovered Bcc species from infected CF patients (Zlosnik et al. 2020; Kenna et al. 2017). The intrinsic resistance of Bcc strains to most antibiotics (Lauman and Dennis 2021) further complicates their eradication.

Bcc bacteria possess large genomes (ranging from approximately 6.4 to 9.0 Mb), composed by two to three replicons and a variable number of plasmids. A total of 2293 Bcc genomes sequenced were publicly available by 13 March 2024 in the National Center for Biotechnology Information (NCBI,https://www.ncbi.nlm.nih.gov/datasets/genome/?taxon=87882). The availability of this impressive number of Bcc genomes allows their exploitation, facilitating post-genomics studies, namely the identification of genes of interest that could be used as targets for the development of novel strategies to prevent Bcc pathogenicity towards CF patients. Despite the impressive number of Bcc available genomes, the majority of post-genomics studies use the genome of *B. cenocepacia* J2315 as reference, mainly due to the fact that it is one of the most virulent Bcc strains known, its involvement in outbreaks and cases of patient-to-patient transmission (Govan and Deretic 1996). Furthermore, *B. cenocepacia* J2315 was the first Bcc genome publicly available (Holden et al. 2009).

## Bacterial sRNAs

Bacterial pathogenesis is complex, and results not only from the expression of a single gene, but often involves the expression of multiple genes, in a tightly and highly regulated manner. Gene expression can be regulated at both the transcription and post-transcriptional levels. A growing body of evidence has highlighted small non-coding RNAs (sRNAs) as major players of post-transcriptional regulation of gene expression in bacteria. sRNAs can be described as RNA molecules that are transcribed from non-canonical start codons, do not encode for proteins, are not components of ribosomes, and are not amino acid residues carriers for protein synthesis. However, about 11 bacterial sRNAs were found to contain open reading frames that encode small proteins, defining a new class known as dual-function sRNAs (Schnoor et al. 2023). In general, sRNA molecules range from about 50 to 250 nucleotides in length (Sharma and Vogel 2009), with numerous exceptions. Bacterial genomes encode variable numbers of these RNA molecules. In the case of *Escherichia coli*, almost a thousand sRNAs were estimated to be encoded in its genome (Saetrom et al. 2005), but the total of *E. coli* sRNAs experimentally demonstrated to be transcribed are far below this number.

**Fig. 1:**
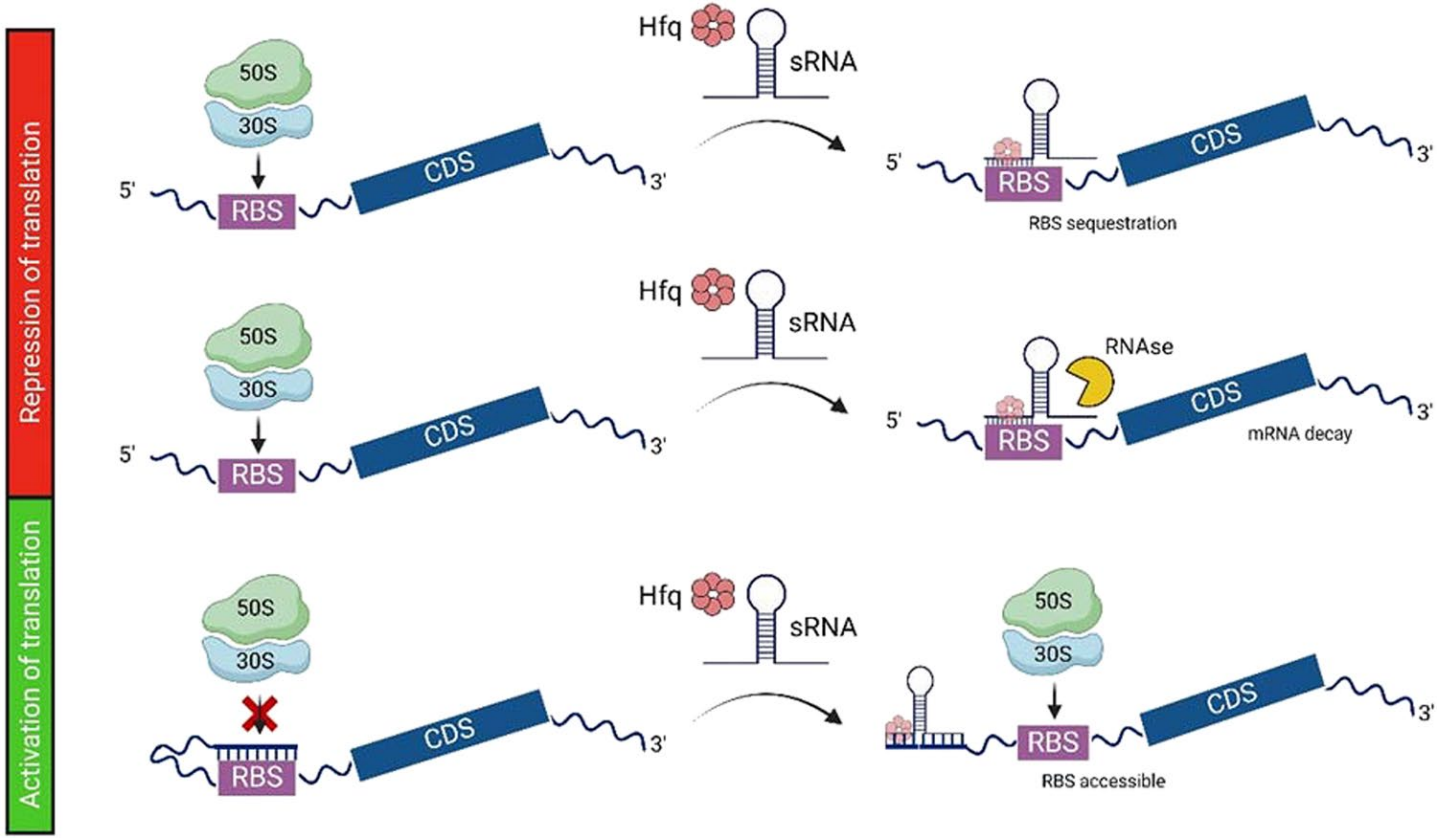
Main mechanisms of sRNA-mRNA interaction. The interaction of sRNAs with their target mRNA can result in the repression or activation of the translation. In the case of translation repression, the sRNA binds to the mRNA Ribosome Binding Site (RBS) region, and translation is inhibited (upper panel). This interaction can lead to the restructuring of the mRNA, exposing nucleolytic sites. RNase E has been reported to be part of the complex made by the sRNA, the mRNA, and the chaperone Hfq (central panel). Activation of translation occurs when the secondary structure adopted by the mRNA does not allow its translation. When this inactive mRNA interacts with the sRNA, in a region usually close to the 5´end, the mRNA secondary structure is rearranged, the RBS becomes exposed, and translation can occur (lower panel). CDS, coding sequence. Created with BioRender.com (license number ST26KPJ2S6)

Most of the known sRNAs exert their regulatory action by base-pairing with mRNAs (designated as the mRNA targets), modulating their stability (Vogel and Wagner 2007). A few sRNAs are known to bind to proteins, sequestering them and inhibiting their activity (Storz et al. 2005). The interaction between sRNAs and mRNAs is driven by the complementary between the two molecules, with a required minimum of perfect match of seven sequential nucleotides, known as the seed region (Wagner and Simons 1994). In bacteria, most of these interactions are promoted by RNA chaperones, such as the Hfq and the less studied ProQ (Melamed et al. 2010). Considering the genomic location relative to their mRNA targets, sRNAs can be classified as cis- or trans-encoded. The so-called cis sRNAs are encoded in the opposite strand of their targeted mRNA, therefore exhibiting a high degree of homology and the interaction with the mRNA target can occur without the mediation of a RNA chaperone. Since known cis-encoded sRNAs only target the mRNA encoded in the DNA strand complementary to the one where they are encoded, in this work, we will not further discuss this class of sRNAs. In fact, trans-encoded sRNA are the best studied sRNAs, are encoded in intergenic regions, and could target multiple mRNAs transcribed from chromosomal loci distinct from that encoding the sRNA, thus acting as global regulators. This class of sRNAs exhibit a limited sequence homology to their mRNA target, and RNA chaperone mediation is required to promote their interaction with the mRNA targets. Hfq is the best studied sRNA-mRNA mediator, and a Hfq-like encoding gene has been found in about half of the bacterial genomes sequenced (Valentin-Hansen et al. 2004). Bacteria of the *Burkholderia cepacia* complex are an exception, with two non-identical copies encoded in their genomes (Sousa et al. 2010). Studies in *E. coli* and *Salmonella* have shown that Hfq forms a hexamer with a donut-like shape, promoting the encounter of sRNAs and mRNA, but not taking part on the interaction (Schumacher et al. 2002). Most of these sRNA-mRNA interactions lead to the decay of the mRNA molecule with the consequent negative regulation of gene expression, although there are some examples of stabilization and therefore an active regulation of gene expression (Fig. 1, lower panel). In the case of negative regulation, the binding of the sRNA usually occludes the ribosome binding site, impeding the translation of the mRNA (Fig. 1, upper panel). The binding of sRNA often leads to the restructuring of the mRNA and exposure of nucleolytic sites, leading to a faster decay of the mRNA due to the action of RNases such as RNase E (Fig. 1, central panel). The RNase activity has also been reported in some cases as part of the complex Hfq-sRNA-mRNA, and it has been described that in some cases the interaction can result in a fast decay of the messenger (Fig. 1, central panel).

### Identification and functional characterization of sRNAs in Bcc bacteria

The first systematic bioinformatics search for trans-encoded sRNAs in a Bcc genome was performed by Coenye et al. (2007). The authors were able to identify 213 putative sRNAs from *B. cenocepacia* J2315, based on comparative genomics and secondary structure predictions. From these 213 putative sRNA, four were experimentally demonstrated to be expressed. A very low degree of conservation of the sRNA sequences was found, even within closely related species. Despite the identification of these putative sRNAs, their functions remained to be elucidated.

One of the characteristics of bacterial sRNAs is that they are transiently expressed, i.e., most of the sRNAs are only expressed under specific conditions, usually as a response to a specific stressor (Rau et al. 2015). Therefore, many research groups have performed sRNA identification based on combined experimental and bioinformatics approaches, using specific environmental conditions, including among others for Bcc bacteria, oxidative stress, low oxygen, iron deprivation, and biofilm mode of growth (Sass et al. 2017; Sass et al. 2015). These conditions were selected since they mimic the environment faced by Bcc when colonizing/infecting the CF lung. These advances have been possible due to technical advances in experimental approaches such as transcriptomics, tiling arrays, co-immunoprecipitation, and deep-sequencing or RNA-seq, among others (Altuvia 2007), as well as due to the development of bioinformatics tools dedicated to RNA. This is the case of most of the sRNAs identified in the Bcc.

Aiming at the unveiling of the molecular mechanisms underlying the resistance of *B. cenocepacia* J2315 against reactive oxygen species when growing as biofilms, Peeters et al. (2010) used custom-made microarrays, containing protein-encoding genes and 1520 probes for selected intergenic regions (IG), to analyze the transcriptomic responses of sessile cells upon exposure of high concentrations of hydrogen peroxide or sodium hypochlorite. Besides the identification of several upregulated genes related to resistance to oxidative stress, DNA repair and other physiological responses were also identified. A total of 39 and 56 of the 1520 IGs were found as upregulated upon hydrogen peroxide or hypochlorite treatment, respectively. Fifty-four and 68 IGs were downregulated under the same conditions. Some of the IGs whose transcription levels were affected by the oxidative stress inducing agents were previously identified under distinct conditions, such as growth in cystic fibrosis (CF) sputum (Drevinek et al. 2008). However, the mechanisms of action and molecular targets of the identified sRNAs were not determined, except for an IG transcript with a secondary structure resembling the 6S RNA consensus structure. In *E. coli*, the 6S RNA is involved in competitive survival in stationary phase and in survival in long-term stationary phase in noncompetitive growth, as well as the regulation of factors such as Crp, FNR, ppGpp, and general translation machinery (Wassarman 2018).

Using an experimental approach based on co-purification of the RNA chaperone Hfq with small-sized RNA extracted and purified from *B. cenocepacia* cells grown in LB medium, followed by cDNA synthesis, cloning sequencing and bioinformatics analysis of sequences, Ramos et al. (2013) found 24 sRNAs that escaped previous bioinformatics and transcriptomics analyses, highlighting the importance of the specific experimental conditions to identify sRNAs in bacteria. The sRNAs were found as unevenly distributed among the *B. cenocepacia* J2315 chromosomes, similar to what has been found by others. None of the identified sRNAs were functionally characterized.

Sass et al. (2015) used a genome-wide analysis transcriptomic analysis to unveil sRNAs expressed by *B. cenocepacia* J2315 grown under biofilm conditions. A total of 15 sRNAs were found to be conserved among *Burkholderia* species and highly abundant in cells growing as biofilms compared with planktonic cells. Although the function of these sRNAs was not unveiled in this work, the authors suggest that they might be involved in adaptation to nutrient limitation and growth arrest. The majority of the sRNAs were predicted to be involved in carbon metabolism. Three of the identified sRNAs were later functionally characterized, NcS25, NcS27, and NcS35. The NcS25 was found to be a strong negative regulator of the porin BCAL3473, involved in the transport of arginine, tyrosine, tyramine, and putrescine across the *B. cenocepacia* J2315 outer membrane (Sass and Coenye 2023). This porin plays an important role in the nitrogen metabolism of *B. cenocepacia* J2315. The sRNA NcS27 was found as conserved among members of the Burkholderiales order (Sass et al. 2019). The sRNA was predicted to target genes involved in transport and metabolism of amino acids and carbohydrates. The overexpression of the sRNA NcS27 was found to attenuate the bacterial growth on several substrates including phenylalanine, tyrosine, glycerol, and galactose, with no effects when bacteria were grown on other substrates. The functional characterization of NcS27 further revealed that the sRNA affected the expression of numerous predicted targets, including genes involved in phenylalanine and tyrosine catabolism, and carbohydrates transport. The authors pointed out NcS27 as a regulator of metabolism shutdown upon nutrient deprivation. The sRNA NcS35, found as playing a role in biofilm formation, was also functionally characterized by Kiekens et al. (2018). This sRNA was highly expressed when *B. cenocepacia* J2315 grow in biofilms and in minimal medium. This sRNA was found to be involved in slowing bacterial growth and metabolism, being therefore hypothesized to protect *B. cenocepacia* J2315 against stressors and enhancing bacterial survival under non-favorable conditions.

Ghosh et al. (2017) sequenced the genome of *B. cenocepacia* KC-01, identified in silico various putative sRNAs, and experimentally confirmed the expression of seven of them, named Bc_KC_sr1 to Bc_KC_sr7. Under growth conditions of iron depletion, the sRNAs Bc_KC_sr1 and Bc_KC_sr2 were upregulated. Two other sRNAs, Bc_KC_sr3 and Bc_KC_sr4 were responsive to medium supplementation with 60 µM hydrogen peroxide. Expression of Bc_KC_sr2, Bc_KC_sr3, and Bc_KC_sr4 was also altered when changing temperature and incubation time. Data base searches indicate Bc_Kc_sr5 and Bc_KC_sr6 as tmRNA and 6S RNA, respectively. Although the functional characterization of the sRNAs was not carried out, the bioinformatics studies led the authors to suggest that the identified sRNAs play a role in the biosynthesis of Fe–S clusters and siderophore, oxidative stress defence, acting as transcription and translation regulators.

The sRNA BrrF was found as overexpressed in *Burkholderia cenocepacia* J2315, when grown under conditions of iron depletion (Sass and Coenye 2020). The predicted targets of this sRNA include the iron-containing enzymes of the tricarboxylic acid cycle *acnA*, *fumA*, *sdhA* and *sdhC*, as well as the iron-containing enzymes *sodB* and *katB* involved in oxidative stress defence. The predicted targets were experimentally confirmed. The authors concluded that BrrF is a regulator of central metabolism and oxidative stress response, contributing to iron-sparing and maintenance of iron homeostasis under iron starvation conditions.

Aiming at the identification of sRNAs playing a role in *B. cenocepacia* virulence, Pita et al. (2023) used the nematode *Caenorhabditis elegans* as an infection model and extracted total RNA from bacteria inside the nematode guts after 48 h of infection. The extracted RNA was analyzed by RNA-seq. The authors were able to find a total of 108 new sRNAs and 31 sRNAs previously described by other authors. One sRNA, named RIT11b, was found as downregulated in bacteria infecting *C. elegans*, directly affecting *B. cenocepacia* phenotypes including virulence, biofilm formation, and swimming motility. The authors further showed that the overexpression of RIT11b led to the reduced expression of the *dusA* and *pyrC* targets, previously found as involved in biofilm formation, epithelial cell adherence, and chronic infections in other bacteria. The in vitro direct interaction of RIT11b with the messengers corresponding to *dusA* and *pyrC* was demonstrated. This was the first report of a sRNA directly involved in *B. cenocepacia* virulence that was functionally characterized. Table 1 summarizes the functionally characterized sRNAs from Bcc bacteria.

**Table 1:** sRNAs functionally characterized in *Burkholderia cenocepacia*

sRNA	Length (bp)	Expression	Function	Identification method	Reference
BrrF (NcS63)	126	Upregulated under conditions of iron depletion	Regulator of central metabolism and oxidative stress response under iron starvation conditions	dRNA-seq, 3'RACE, qPCR, Northern blot	(Sass and Coenye 2020)
NcS25	84	Highly expressed in biofilms	Regulates the expression of the outer membrane porin BCAL3473	dRNA-seq, 3'RACE, qPCR, Northern blot	(Sass and Coenye 2023)
NcS27	92	Highly expressed in biofilms and accumulates under growth arrest	Role in balancing the shutdown of metabolism upon nutrient deprivation	dRNA-seq, 3'RACE, qPCR	(Sass et al. 2019)
NcS35	166	Higher expression in biofilms, presence of SDS and in minimal medium M9	Attenuating effect on the metabolic rate and growth	dRNA-seq, 3'RACE, qPCR, Northern blot	(Kiekens et al. 2018)
RIT11b (NcS06)	259	Downregulated under *C. elegans* infection conditions	Pleiotropic regulator involved in *B. cenocepacia* virulence, biofilm formation, and swimming motility	dRNA-seq, Cappable-seq, qPCR, Northern blot	(Pita et al. 2023; Sass et al. 2017)

### The expression of a single sRNA can be affected by distinct stress conditions

In other bacteria, a single sRNA can be found as belonging to more than one regulon (Papenfort and Melamed 2023), and thus one can expect a specific sRNA to be upregulated or downregulated under distinct stress conditions. To unveil Bcc sRNAs putatively involved in response to more than one stress conditions, we retrieved published data on Bcc sRNAs expression under distinct conditions, including infection of *Caenorhabditis elegans*, low oxygen, oxidative stress, low iron, low pH, and growth as biofilm. A total of 34 Bcc sRNAs were identified as differentially expressed under at least two different conditions (Table 2). From the host–pathogen perspective, it is worth to mention that sRNAs RIT23a, RIT31, RIT43, RIT53, RIT77, and RIT90 were found as upregulated in conditions of *Caenorhabditis elegans* infection and under biofilm growth conditions, suggesting the occurrence of common regulatory features in bacterial physiology when adapting to the host and when adopting the biofilm mode of growth. Other examples are RIT1 and RIT81, found as upregulated when the bacteria infect *C. elegans* and when growing under limitation of oxygen and when in the biofilm mode of growth. Data summarized on Table 2 also show examples of opposite responses, as is the case of RIT11b, downregulated when bacteria infect the *C. elegans* and upregulated when growing as a biofilm.

**Table 2:** *B. cenocepacia* sRNAs identified in different studies as expressed under specific conditions

Replicon	Strand	Start	End	Designation	Other designations	Conservation in *B. cenocepacia*^1,2^	Conservation in Bcc^1,2^	Conservation in *B. pseudomallei*^1,2^	*C. elegans* infection	Low O_2_ concentration	Oxidative stress (organic peroxyde)	Oxidative stress (inorganic peroxyde)	Low iron concentration		Low pH		Biofilms
									Cappable-seq^1^	Microarray^3^	Microarray^3^	Microarray^3^	Microarray^3^	qRT-PCR^4^	Microarray^3^	qRT-PCR^4^	dRNA-Seq^4^
Chr 1	+	3109	3211	RIT1	IG1_2776	C	C	NC	Up	Up	-	-	-		-		Expressed*
Chr 1	+	20,581	20,685	RIT2a	NcS01	C	C	SC	-	ND	ND	ND	ND	-	ND	Down	Expressed (Up)
Chr 1	+	269,841	269,946	RIT7	IG1_269746	C	SC	NC	Up	-	Down	-	-	ND	-	ND	Expressed*
Chr 1	+	582,834	583,013	RIT10	IG1_582769	C	NC	NC	Up	Up	-	-	-	ND	-	ND	Not expressed*
Chr 1	+	603,684	604,017	**RIT11b**	ncRNA7; NcS06; BTH_s1	C	C	NC	Down	ND	ND	ND	ND	-	ND	-	Expressed (Up)
Chr 1	+	1,783,299	1,783,698	RIT23a	ncRI8	C	NC	NC	Up	ND	ND	ND	ND	ND	ND	ND	Expressed
Chr 1	+	2,136,329	2,136,454	RIT25	nc5U15; Bc_KC_sr2	C	C	NC	Down	ND	ND	ND	ND	ND	ND	ND	Expressed
Chr 1	+	2,548,559	2,548,732	**NcS63**	BrrF; RIT29a; BTH_s39	C	C	SC	-*	ND	ND	ND	ND	Up	ND	Down	Expressed (Up)
Chr 1	+	2,872,901	2,873,090	RIT31	nc5U26	C	C	NC	Up	ND	ND	ND	ND	ND	ND	ND	Expressed
Chr 1	+	2,912,200	2,912,274	RIT32	NcS16; Bc_KC_sr1	C	C	C	Down	ND	ND	ND	ND	-	ND	-	Expressed
Chr 1	+	2,967,641	2,967,787	RIT34	nc5U30; Bc_KC_sr7; IG1_2967543	C	C	C	Down	-	Up	-	-	ND	-	ND	Expressed
Chr 1	+	3,246,834	3,246,906	RIT39	IG1_3246787	NC	NC	NC	-*	-	Up	Up	-	ND	-	ND	Not expressed*
Chr 1	+	3,596,979	3,597,129	RIT43	nc5U42; IG1_3596870	C	C	NC	Up	Down	Down	Down	-	ND	-	ND	Expressed
Chr 1	+	3,621,767	3,621,868	RIT44	nc5U44; IG1_3621670	C	C	NC	Down	Down	-	-	-	ND	Down	ND	Expressed
Chr 1	-	2,673,443	2,673,333	RIT53	nc5U24	C	C	NC	Up	ND	ND	ND	ND	ND	ND	ND	Expressed
Chr 1	-	2,545,503	2,545,298	RIT55	NcS11; ncRNA13	C	C	C	Down	ND	ND	ND	ND	-	ND	-	Expressed (Up)
Chr 1	-	2,195,731	2,195,546	RIT60	nc5U16	C	SC	NC	-	ND	ND	ND	ND	ND	ND	ND	Expressed
Chr 1	+	3,008,232	3,008,587	RIT98	ncRI12; ncRNA6; IG1_3008003	C	C	C	Up	Down	-	-	-	ND	Up	ND	Expressed
Chr 1	-	444,591	444,034	RIT99	IG1_443956	C	C	C	Up	Down	Up	-	-	ND	Down	ND	Not expressed*
Chr 1	-	42,890	42,693	NcS02	IG1_42625; Bp1_781	C	C	C	Not Expressed	Down	-	-	-	-	-	-	Expressed (Up)
Chr 1	-	3,298,074	3,297,990	**NcS25**	IG1_3297972	SC	C	C	-	-	-	-	-	-	-	-	Expressed (Up)
Chr 1	+	2,935,785	2,936,025	NcS17	ncRNA4; Bc_KC_sr6; IG1_2935724	C	C	C	Not Expressed	-	Up	-	-	ND	-	ND	Expressed
Chr 1	-	3,666,648	3,666,557	**NcS27**		SC	C	NC	-	ND	ND	ND	ND	-	ND	-	Expressed (Up)
Chr 1	+	221,261	221,364	RIT100	NcS03	C	C	SC	Down	ND	ND	ND	ND	-	ND	Up	Expressed (Up)
Chr 2	+	1,106,447	1,106,581	RIT77	BTH_s31; Bp2_287	C	C	NC	Up	ND	ND	ND	ND	ND	ND	ND	Expressed
Chr 2	+	1,665,995	1,666,211	RIT79	ncRI23	C	NC	NC	-*	ND	ND	ND	ND	ND	ND	ND	Expressed
Chr 2	+	1,953,292	1,953,510	RIT81	BTH_s35; IG2_1953120	C	C	NC	Up	Up	-	-	-	ND	-	ND	Expressed
Chr 2	+	2,307,538	2,307,633	RIT82	IG2_2307283	C	SC	NC	Up	Down	Down	-	-	ND	-	ND	Expressed*
Chr 2	+	2,466,930	2,467,069	RIT83	IG2_2466870	SC	SC	NC	Up	Down	-	-	-	ND	-	ND	Expressed*
Chr 2	-	2,917,275	2,917,200	RIT86	ncRI26	C	NC	NC	Down	ND	ND	ND	ND	ND	ND	ND	Expressed
Chr 2	-	1,889,107	1,888,972	RIT90	nc5U54	SC	SC	NC	Up	ND	ND	ND	ND	ND	ND	ND	Expressed
Chr 2	-	2,304,213	2,304,378	**NcS35**		C	C	NC	Up	ND	ND	ND	ND	-	ND	-	Expressed (Up)
Chr 2	+	1,926,664	1,926,802	NcS33	ncRNA11; IG2_1926503	SC	NC	NC	Not Expressed	Down	-	-	-	ND	-	ND	Expressed
Chr 2	+	2,568,766	2,568,918	NcS37		C	SC	NC	Down	ND	ND	ND	ND	-	ND	-	Expressed (-)

sRNAs already characterized in *B. cenocepacia* J2315 are designated in bold; *ND*, no data/not determined; “-”, not differentially expressed; *, inferences made by the authors despite the low quality of the data; *C*, conserved; *SC*, semi-conserved; *NC*, non-conserved. In biofilms conditions, the sRNAs were identify by comparing the differential RNA-Seq dataset to conventional RNA-Seq data, with the results presented as expressed or not expressed. The differential expression of some sRNAs was assessed by qRT-PCR, and the results are indicated in parentheses (Biofilms) or in qRT-PCR columns (conditions of low iron concentration and low pH). Data was retrieved from ^1^Pita et al. (2023), ^2^Pita et al. (2018), ^3^Sass et al. (2013), and ^4^Sass et al. (2017)

### sRNAs from non-Bcc *Burkholderia* spp*.*

sRNAs have also been found in other non-Bcc bacteria, namely from *B. pseudomallei*, the causative agent of melioidosis, clinically presenting as a febrile disease, ranging from a chronic debilitating local infection to acute lethal septicemia with an associated mortality of ten to more than 40% (White 2003). Khoo et al. (2012) developed a pipeline integrating several sRNA prediction bioinformatics tools and found a total of 1306 sRNA putative genes within available genomes of *B. pseudomallei*, 21 of them with homologs in the Rfam database (Kalvari et al. 2021). Only 15 sRNAs were shortlisted due to their conservation in *Burkholderia* spp. or different *B. pseudomallei* strains, and the expression of eight of these sRNAs were experimentally demonstrated to be expressed. None of them was functionally characterized.

Stubben et al. (2014) cultured *B. thailandensis* (a species closely related to *B. pseudomallei*) under 54 distinct growth conditions and used a *Burkholderia*-specific microarray with probes for all intergenic regions greater than 90 bases. This approach allowed the identification of 38 novel sRNA, and the expression of five of them was experimentally validated. The sRNAs BTH_s1 and s39 exhibited differential expression profiles depending on the growth phase, exposure to antibiotics or supplementation with serum. BTH_s39 was demonstrated to affect bacterial metabolism and adaptation to the host.

## Conclusions

**Fig. 2:**
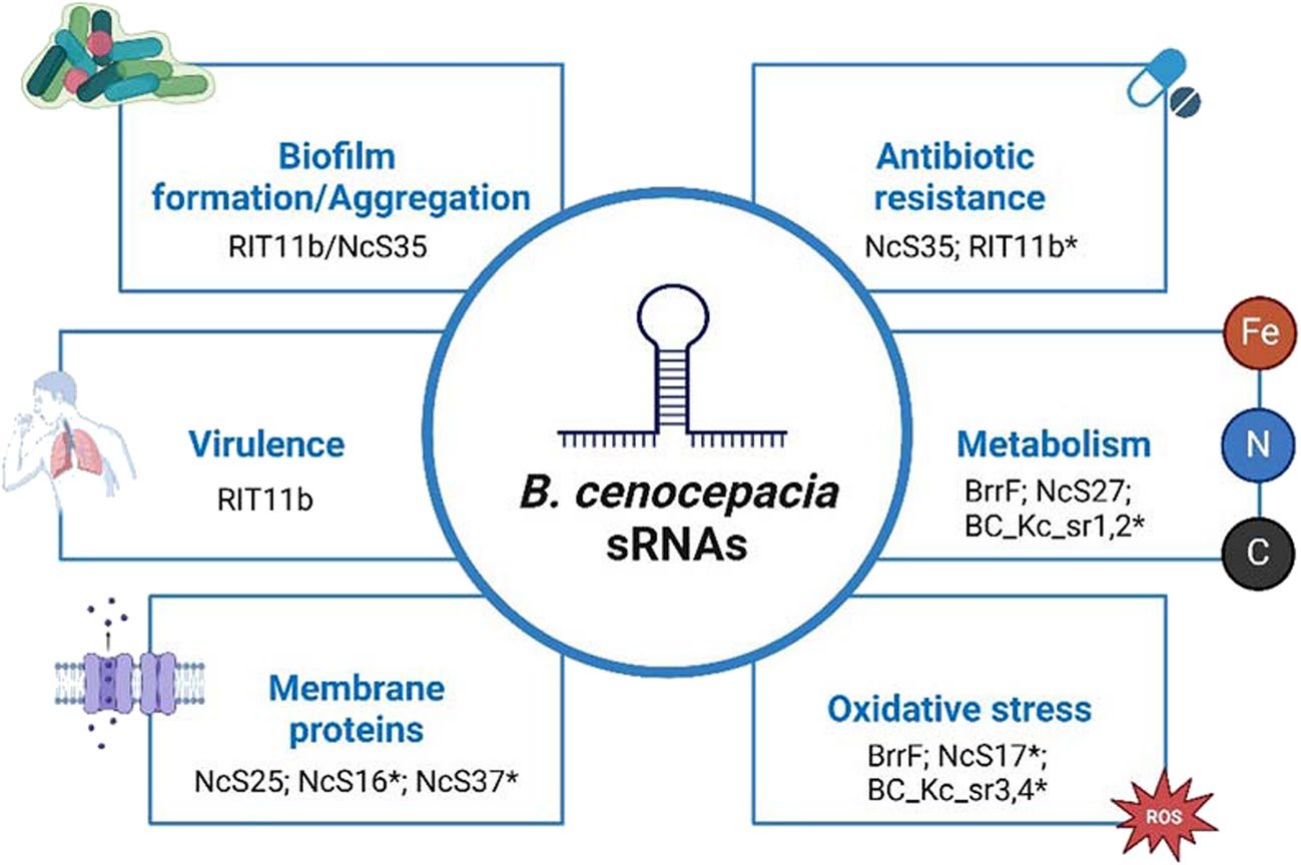
Schematic representation of known sRNAs and their impact on Bcc bacteria. An * indicates a predicted function not fully demonstrated. Fe, N, C: iron, nitrogen, and carbon metabolism. ROS, reactive oxygen species. Created with BioRender.com (license number XF26KPKM64)

Post-transcriptional regulation of gene expression is keen to bacterial opportunistic pathogens like the members of Bcc, allowing its fast, accurate, and successful adaptation to the challenging environment of their hosts. Several sRNAs have been functionally characterized in Bcc and closely related pathogens like the *B. pseudomallei* group. So far, sRNAs are known to regulate several traits of Bcc, impacting their biology and relations with their hosts. These include carbon and nitrogen metabolism, resistance to stresses like oxidative stress, fine tuning of transporters, resistance to antibiotics (JR Feliciano, GR Matos and JH Leitão, unpublished results), and virulence towards the nematode *C. elegans*. A schematic summary of known sRNAs and their impacts in Bcc is depicted in Fig. 2.

Regardless of the already known functions of sRNAs, the number of sRNAs with unknown functions in Bcc bacteria remains amazingly high. Future research on this topic will certainly add further clarity to our knowledge on the biology and gene expression regulation of these highly versatile and adaptable bacteria. This new knowledge will also bring the opportunity to design novel sRNA-based strategies to control the pathogenicity of Bcc bacteria.

## Data Availability

All data generated or analyzed during this study are included in this published article.
